# Supplementary material for: Origin of imported SARS-CoV-2 strains in The Gambia identified from whole genome sequences
Source: PLoS One. 2021 Aug 31;16(8):e0241942. doi: 10.1371/journal.pone.0241942 (PMC8407536; doi:10.1371/journal.pone.0241942)
Supplement: Attachment — Submitted filename: Response to Reviewers.docx [file pone.0241942.s001.docx]

Medical Research Council Unit The Gambia at LSHTM

P.O Box 273

Banjul, The Gambia

Dear sir,

I wish to thank you for your time and effort in reviewing our manuscript title “Origin of imported SARS-CoV-2 strains in The Gambia identified from whole genome sequences submitted in your Journal. The authors have thoroughly thought about all the major comments made on the manuscript and decided to change the paper to a short communication. Please find below answers (in red) to the concerns highlighted by the reviewers.

Journal Requirements:

When submitting your revision, we need you to address these additional requirements.

1. Please ensure that your manuscript meets PLOS ONE's style requirements, including those for file naming. The PLOS ONE style templates can be found at

[https://journals.plos.org/plosone/s/file?id=wjVg/PLOSOne_formatting_sample_main_body.pdf](https://protect-eu.mimecast.com/s/VZ19Cwr14SLWg9JSqKMLB?domain=journals.plos.org) and

[https://journals.plos.org/plosone/s/file?id=ba62/PLOSOne_formatting_sample_title_authors_affiliations.pdf](https://protect-eu.mimecast.com/s/igjeCx1g4cJ3PwniYmIz2?domain=journals.plos.org)

 I have formatted the manuscript using the guidelines highlighted above.

2. We note that you are reporting an analysis of a microarray, next-generation sequencing, or deep sequencing data set. PLOS requires that authors comply with field-specific standards for preparation, recording, and deposition of data in repositories appropriate to their field. Please upload these data to a stable, public repository (such as ArrayExpress, Gene Expression Omnibus (GEO), DNA Data Bank of Japan (DDBJ), NCBI GenBank, NCBI Sequence Read Archive, or EMBL Nucleotide Sequence Database (ENA)). In your revised cover letter, please provide the relevant accession numbers that may be used to access these data. For a full list of recommended repositories, see http://journals.plos.org/plosone/s/data-availability#loc-omics or http://journals.plos.org/plosone/s/data-availability#loc-sequencing

The genomes are now deposited at GenBank with the following submission number: SUB9545765

3. Please provide additional details regarding participant consent.

In the ethics statement in the Methods and online submission information, please ensure that you have specified (i) whether consent was informed and (ii) what type you obtained (for instance, written or verbal, and if verbal, how it was documented and witnessed). If your study included minors, state whether you obtained consent from parents or guardians. If the need for consent was waived by the ethics committee, please include this information.

If you are reporting a retrospective study of medical records or archived samples, please ensure that you have discussed whether all data were fully anonymized before you accessed them and/or whether the IRB or ethics committee waived the requirement for informed consent. If patients provided informed written consent to have data from their medical records used in research, please include this information.
Once you have amended this/these statement(s) in the Methods section of the manuscript, please add the same text to the “Ethics Statement” field of the submission form (via “Edit Submission”).

For additional information about PLOS ONE ethical requirements for human subjects research, please refer to [http://journals.plos.org/plosone/s/submission-guidelines#loc-human-subjects-research](http://protect-eu.mimecast.com/s/1H9RCyXjgiNRAkqCROiAO?domain=journals.plos.org).

 This is sorted accordingly

4. Your ethics statement should only appear in the Methods section of your manuscript. If your ethics statement is written in any section besides the Methods, please move it to the Methods section and delete it from any other section. Please ensure that your ethics statement is included in your manuscript, as the ethics statement entered into the online submission form will not be published alongside your manuscript.

[Note: HTML markup is below. Please do not edit.]

Reviewers' comments:

Reviewer's Responses to Questions

**Comments to the Author**

1. Is the manuscript technically sound, and do the data support the conclusions?

The manuscript must describe a technically sound piece of scientific research with data that supports the conclusions. Experiments must have been conducted rigorously, with appropriate controls, replication, and sample sizes. The conclusions must be drawn appropriately based on the data presented.

Reviewer #1: Yes

Reviewer #2: Partly

Reviewer #3: Partly

Reviewer #4: Yes

2. Has the statistical analysis been performed appropriately and rigorously?

Reviewer #1: Yes

Reviewer #2: N/A

Reviewer #3: No

Reviewer #4: N/A

3. Have the authors made all data underlying the findings in their manuscript fully available?

The [PLOS Data policy](http://protect-eu.mimecast.com/s/VC7yCzXkjiRAO9QuoXvx_?domain=plosone.org) requires authors to make all data underlying the findings described in their manuscript fully available without restriction, with rare exception (please refer to the Data Availability Statement in the manuscript PDF file). The data should be provided as part of the manuscript or its supporting information, or deposited to a public repository. For example, in addition to summary statistics, the data points behind means, medians and variance measures should be available. If there are restrictions on publicly sharing data—e.g. participant privacy or use of data from a third party—those must be specified.

Reviewer #1: Yes

Reviewer #2: Yes

Reviewer #3: No

Reviewer #4: No

4. Is the manuscript presented in an intelligible fashion and written in standard English?

PLOS ONE does not copyedit accepted manuscripts, so the language in submitted articles must be clear, correct, and unambiguous. Any typographical or grammatical errors should be corrected at revision, so please note any specific errors here.

Reviewer #1: Yes

Reviewer #2: Yes

Reviewer #3: Yes

Reviewer #4: Yes

5. Review Comments to the Author

Please use the space provided to explain your answers to the questions above. You may also include additional comments for the author, including concerns about dual publication, research ethics, or publication ethics. (Please upload your review as an attachment if it exceeds 20,000 characters)

Reviewer #1: Dear author(s),
I have read your submission and it was interesting. Only there are minor things that you should consider for having better insight to your study. You may pay attention to this point that whether or not this study has been done previously by others and compare your results. Another point that you should consider is that what is your main goal for future of performing this work and what was your expectation out of that data.

Reviewer #2: In this paper, Kanteh et al. reported whole genome sequence of SARS-CoV-2 strains detected in Gambia and their phylogenetic analysis with other strains outside of the country. Given little viral sequence information from African countries, the study provides us with a clue to elucidate the global transmission dynamics of the virus at the relatively early time point of the current outbreak. However, I have several concerns in the manuscript to be addressed before the publication.

Major points
1. Absolute “origin” or “source” cannot be inferred from phylogenetic tree. That is because there must be other viral strains from other countries that are more closely related to Gambian strains but unsampled and not included in their phylogenetic analysis.

The reviewer is very right about this point. However, at the time of analysis, we ran all the sequences against the available genomes on Nextclade (n=4645) and constructed a phylogenetic tree. Despite the number of genomes added, the origin or source still remains the same as seen in the image below.

2. Related to the point #1, viral sequences from other countries included in the phylogenetic analysis are very few. There should be many more available sequence data of viral strains collected by March 2020 from all over the world in GISAID, ViPR, and so on.

Yes, this is true. We actually ran our samples against other genomes from Nexstrain, however, we still have similar topology. Selection of genomes from GISAID was based on our pateint’s travel history.

3. Difference in sequences between Illumina and Nanopore protocols should be done by not phylogenetic trees but direct comparison of SNPs (number and positions) between them.

I agree with you. However, if there was huge difference in terms of the number of SNPs from each platform, this would perhaps alter the topology of the tree. However, this was not seen except in one sample, where the Illumina data had a longer branch length that the Nanopore. We found that there were more SNPs from the Illumina than the Nanopore data.

4. Related to the point #3, the authors had better show either of only Figure 2 or Figure

Given that both trees look similar, we decided to include only one in the manuscript. We also added a phylogenetic tree showing all the genomes in Nextclade between march and April 2020.

Minor points
5. There are a lot of typo in the manuscript. Please read the manuscript carefully and correct them before re-submission.

This has been corrected.

6. (Lines 34 and 203) “Wuhan reference genome” is not a scientific term. Please indicate the actual strain name as described in the line 177.

Okay. This is sorted.

7. Texts in the lines 76 and 77-78 are redundant.

This is sorted accordingly.

8. The names of genes for the screening and confirmation should be specified here (as described in the Table 2.)

9. I personally think Figure 1 is unnecessary and can be removed from the manuscript. Yet, I will leave the decision to the editor and authors.

10. In the table 2, there is “Ct” in the third column title. However, I do not see any Ct values in the table.

11. (Line 249) WGS should be spelled out.

Reviewer #3: The authors conducted complete genomic sequencing of the first 6 cases of SARS-CoV-2 in The Gambia to determine/confirm their origin/genetic characteristics using Illumina MiSeq and Nanopore platforms. The study addresses an important issue because the knowledge on the genetic characteristic of SARS-CoV-2 strains circulating in The Gambia/Adrica. The study also appears to be technically sound.
The results presented/discussed are insufficient for a full-length research article. If no additional analysis (see below) is done, the submission must be converted to a short communication.
Overall, the manuscript is too focused on technical details of both sequencing methods employed and presents very little detail on the genomic/molecular analysis of the Gambian SARS-CoV-2 strains. This has to be changed by mostly re-writing the manuscript.
It is not clear why the two sequencing approaches had to be used: Illumina MiSeq and Nanopore. Although, the authors stated that both methods had their own advantages, it is not clear why the two methods were needed to just determine the strain origin. “While Illumina sequencing may be more accurate in determining within sample-diversity, Nanopore data can help with the understanding of the linkage between SNPs within individual virions”. The importance/relevance of determining within host/virion diversity in this study is not clear. It is well established fact that coronaviruses exist as quasispecies within the same host.
The abstract needs to be re-written. Currently, it’s a mere statement of the methodology used in the study. It has to be re-written to properly convey the problem/background, approach, findings and conclusion of the study.
Accession numbers for the newly generated sequences must be provided, but were missing.
Issues with the analysis:
More SARS-CoV-2 strains should be included in the phylogenetic trees.
Phylogenetic distance and bootstrap values should be reflected on the trees.
The meaning of the dashed lines should be explained in the figure legends.
Recombination analysis should be conducted.

Reviewer #4: This is a nice short paper on sequencing some SARS-CoV-2 genomes from Gambia. their methods seem fine, and the sequences seem reasonable. My only concern is that they are not deposited to GenBank. I can't access them from GISAID without permissions to their web pages.

# The genomes are now available at GenBank with the submission: SUB9545765

6. PLOS authors have the option to publish the peer review history of their article ([what does this mean?](https://protect-eu.mimecast.com/s/Bkm0CA1pkc9zG2LsMzH5f?domain=journals.plos.org)). If published, this will include your full peer review and any attached files.


If you choose “no”, your identity will remain anonymous but your review may still be made public.


**Do you want your identity to be public for this peer review?** For information about this choice, including consent withdrawal, please see our [Privacy Policy](https://protect-eu.mimecast.com/s/5CKmCB6q0UVOzBptvzn1F?domain=plos.org).

Reviewer #1: **Yes:** Leila Mousavizadeh

Reviewer #2: No

Reviewer #3: **Yes:** Anastasia Vlasova

Reviewer #4: No

[NOTE: If reviewer comments were submitted as an attachment file, they will be attached to this email and accessible via the submission site. Please log into your account, locate the manuscript record, and check for the action link "View Attachments". If this link does not appear, there are no attachment files.]

While revising your submission, please upload your figure files to the Preflight Analysis and Conversion Engine (PACE) digital diagnostic tool, [https://pacev2.apexcovantage.com/](https://protect-eu.mimecast.com/s/DLR1CD8vnCBkD0NCBZwUf?domain=pacev2.apexcovantage.com). PACE helps ensure that figures meet PLOS requirements. To use PACE, you must first register as a user. Registration is free. Then, login and navigate to the UPLOAD tab, where you will find detailed instructions on how to use the tool. If you encounter any issues or have any questions when using PACE, please email PLOS at [figures@plos.org](mailto:figures@plos.org). Please note that Supporting Information files do not need this step.
