# Supplementary material for: Origin of imported SARS-CoV-2 strains in The Gambia identified from whole genome sequences
Source: PLoS One. 2021 Aug 31;16(8):e0241942. doi: 10.1371/journal.pone.0241942 (PMC8407536; doi:10.1371/journal.pone.0241942)
Supplement: Attachment — Submitted filename: response to Reviewers.docx [file pone.0241942.s002.docx]

| **Date:** | May 28 2021 04:04AM |
| --- | --- |
| **To:** | "Abdoulie Kanteh" akanteh@mrc.gm |
| **From:** | "PLOS ONE" plosone@plos.org |
| **Subject:** | PLOS ONE Decision: Revision required [PONE-D-20-33232R1] |

PONE-D-20-33232R1
Origin of imported SARS-CoV-2 strains in The Gambia identified from whole genome sequences
PLOS ONE

Dear Dr. Kanteh,

Thank you for submitting your manuscript to PLOS ONE. After careful consideration, we feel that it has merit but does not fully meet PLOS ONE’s publication criteria as it currently stands. Therefore, we invite you to submit a revised version of the manuscript that addresses the points raised during the review process.

==============================
The revised manuscript was reviewed by 2 original reviewers. Both still identified serious problems in your revision.  Please review the attched comments and provide carefully conceived responses.
==============================

Please submit your revised manuscript by Jul 12 2021 11:59PM. If you will need more time than this to complete your revisions, please reply to this message or contact the journal office at plosone@plos.org. When you're ready to submit your revision, log on to https://www.editorialmanager.com/pone/ and select the 'Submissions Needing Revision' folder to locate your manuscript file.

Please include the following items when submitting your revised manuscript:

- A rebuttal letter that responds to each point raised by the academic editor and reviewer(s). You should upload this letter as a separate file labeled 'Response to Reviewers'.
- A marked-up copy of your manuscript that highlights changes made to the original version. You should upload this as a separate file labeled 'Revised Manuscript with Track Changes'.
- An unmarked version of your revised paper without tracked changes. You should upload this as a separate file labeled 'Manuscript'.

If you would like to make changes to your financial disclosure, please include your updated statement in your cover letter. Guidelines for resubmitting your figure files are available below the reviewer comments at the end of this letter.

If applicable, we recommend that you deposit your laboratory protocols in protocols.io to enhance the reproducibility of your results. Protocols.io assigns your protocol its own identifier (DOI) so that it can be cited independently in the future. For instructions see: <http://journals.plos.org/plosone/s/submission-guidelines#loc-laboratory-protocols>. Additionally, PLOS ONE offers an option for publishing peer-reviewed Lab Protocol articles, which describe protocols hosted on protocols.io. Read more information on sharing protocols at <https://plos.org/protocols?utm_medium=editorial-email&utm_source=authorletters&utm_campaign=protocols>.

We look forward to receiving your revised manuscript.

Kind regards,

Yury E Khudyakov, PhD
Academic Editor
PLOS ONE


[Note: HTML markup is below. Please do not edit.]

Reviewers' comments:

Reviewer's Responses to Questions

**Comments to the Author**

1. If the authors have adequately addressed your comments raised in a previous round of review and you feel that this manuscript is now acceptable for publication, you may indicate that here to bypass the “Comments to the Author” section, enter your conflict of interest statement in the “Confidential to Editor” section, and submit your "Accept" recommendation.

Reviewer #2: (No Response)

Reviewer #4: (No Response)

2. Is the manuscript technically sound, and do the data support the conclusions?

The manuscript must describe a technically sound piece of scientific research with data that supports the conclusions. Experiments must have been conducted rigorously, with appropriate controls, replication, and sample sizes. The conclusions must be drawn appropriately based on the data presented.

Reviewer #2: Partly

Reviewer #4: Yes

3. Has the statistical analysis been performed appropriately and rigorously?

Reviewer #2: N/A

Reviewer #4: Yes

4. Have the authors made all data underlying the findings in their manuscript fully available?

The [PLOS Data policy](http://www.plosone.org/static/policies.action#sharing) requires authors to make all data underlying the findings described in their manuscript fully available without restriction, with rare exception (please refer to the Data Availability Statement in the manuscript PDF file). The data should be provided as part of the manuscript or its supporting information, or deposited to a public repository. For example, in addition to summary statistics, the data points behind means, medians and variance measures should be available. If there are restrictions on publicly sharing data—e.g. participant privacy or use of data from a third party—those must be specified.

Reviewer #2: Yes

Reviewer #4: No

5. Is the manuscript presented in an intelligible fashion and written in standard English?

PLOS ONE does not copyedit accepted manuscripts, so the language in submitted articles must be clear, correct, and unambiguous. Any typographical or grammatical errors should be corrected at revision, so please note any specific errors here.

Reviewer #2: Yes

Reviewer #4: Yes

6. Review Comments to the Author

Please use the space provided to explain your answers to the questions above. You may also include additional comments for the author, including concerns about dual publication, research ethics, or publication ethics. (Please upload your review as an attachment if it exceeds 20,000 characters)

Reviewer #2: The revised manuscript has been improved to be concise and easy to follow. Still, I have three big concerns in the revised manuscript.

Lines 95-99:
Their finding that 7 SNPs that were observed by Illumina but not by Nanopore appeared only after 10 days of infection in the same individual seem surprisingly a big number to me. The authors argued that "The SNP winked by the Nanopore, might be due to higher accuracy on Illumina..." How could the authors exclude the possibility that there was something wrong in the Illumina run for the particular sample?

Response:

I agree with you, and this was a concern to authors as well. Given the cost involved in sequencing using Illumina, we were not able to re-run the sample to confirm this. To this end, I concur with your argument and added a statement highlighting that this may as well be a sequencing error or issues with the sample.

Figure 1 and texts:
Case F was totally missed in the tree and main texts.

Response:

Case F was a negative Covid sample and hence was used as a negative control. The sample was run through all the processes from start to end, however it was dropped during phylogenetic analysis. This is because the sequences were more of human than SARS-CoV-2 which will affect the alignment as well as the tree. This is now explained clearly in the main text.

Figure 2 and texts:
Although the authors included and indicated 3 Gambian sequences in the tree, they should include and show the sequences of all 6 cases. Besides, there is no explanation about the figure in the main texts at all.

Response:

This is very true. This is now corrected and a phylogenetic tree showing all cases are now included in the main text. We reported 6 cases, however, one of them is a negative sample which is not included in the phylogenetic tree.

Reviewer #4: I still can't find the sequences....

"The genomes are now deposited at GenBank with the following submission number: SUB9545765"

SUB9545765 is not an accession number. I went to the NCBI pages, and did a search against all of the databases, and could not find "SUB9545765" in any of their databases.

The NCBI SARS-CoV-2 portal lives here:
https://www.ncbi.nlm.nih.gov/sars-cov-2/

and there's about a half-million genomes in there, and here the accession number usually contains two letters, followed by six numbers - for example: FR988027 or OU032008 are both accession numbers.

Another example, from a whole genome sequencing project looks like this:
WGS of SARS-CoV-2 circulating in Spain
GenBank Accession: ERX5596932
BioProject ID: PRJEB43166
SRA study link: ERP127101
SRA run number: ERR5956411

Response:

Thanks for highlighting this. After successfully submitting the genomes to NCBI, a submission ID was assigned to my submission. However, each genome was assigned an accession number which I highlighted in the main text in bracket under “**Data and Materials Availability”** The accessions numbers in this case starts with MZ followed by six digits. I apologise for the confusion.

7. PLOS authors have the option to publish the peer review history of their article ([what does this mean?](https://journals.plos.org/plosone/s/editorial-and-peer-review-process#loc-peer-review-history)). If published, this will include your full peer review and any attached files.


If you choose “no”, your identity will remain anonymous but your review may still be made public.


**Do you want your identity to be public for this peer review?** For information about this choice, including consent withdrawal, please see our [Privacy Policy](https://www.plos.org/privacy-policy).

Reviewer #2: No

Reviewer #4: **Yes:** David Ussery

[NOTE: If reviewer comments were submitted as an attachment file, they will be attached to this email and accessible via the submission site. Please log into your account, locate the manuscript record, and check for the action link "View Attachments". If this link does not appear, there are no attachment files.]

While revising your submission, please upload your figure files to the Preflight Analysis and Conversion Engine (PACE) digital diagnostic tool, <https://pacev2.apexcovantage.com/>. PACE helps ensure that figures meet PLOS requirements. To use PACE, you must first register as a user. Registration is free. Then, login and navigate to the UPLOAD tab, where you will find detailed instructions on how to use the tool. If you encounter any issues or have any questions when using PACE, please email PLOS at [figures@plos.org](mailto:figures@plos.org). Please note that Supporting Information files do not need this step.
